# A retrospective preliminary histomorphometric and clinical investigation on sinus augmentation using enzyme-deantigenic, collagen-preserving equine bone granules and plasma rich in growth factors

**DOI:** 10.1186/s40729-021-00336-9

**Published:** 2021-06-11

**Authors:** Danilo Alessio Di Stefano, Raffaele Vinci, Paolo Capparè, Enrico Felice Gherlone

**Affiliations:** 1 Dental School, Vita-Salute University IRCCS San Raffaele, Milan, Italy; 2 Private Practice, Milan, Italy

**Keywords:** Enzyme deantigenic equine bone, PRGF, Histomorphometry, Bone graft, Sinus augmentation

## Abstract

**Background:**

Enzyme-deantigenic equine bone (EDEB) is a substitute of autogenous bone. Mixing it with plasma rich in growth factors (PRGF) seems a viable option to achieve enhanced bone formation in alveolar bone augmentation surgeries. This retrospective study aims to first report the histomorphometric and clinical outcomes achieved when using the EDEB/PRGF mixture for performing sinus augmentation procedures followed by delayed implant placement.

**Materials and methods:**

Records of 11 patients who underwent 14 sinus augmentation surgeries using EDEB/PRGF followed by delayed implant placement were retrospectively collected and analyzed to assess histomorphometric data concerning newly formed bone (NFB) and residual biomaterial (RB) recorded at implant placement, marginal bone loss (MBL) values of implants placed in the augmented sinuses, and implant and prosthetic success and survival rates.

**Results:**

At 5.6 ± 1.1 months after grafting, NFB and RB were 34.0 ± 9.1% and 11.3 ± 2.2% respectively, and no histologic signs of inflammation or immune reaction were observed in any of the 34 bone biopsies being collected. Further, 86.5 ± 4.3 months after implant placement, MBL was 0.40 ± 0.07 mm. No implant or prosthesis failed, and the implant success and survival rates were 100%

**Conclusions:**

Within the limitations of the present study, grafting EDEB/PRGF for lateral sinus augmentation and delayed implant placement seems to be safe. Compared to published data concerning EDEB alone, results of the present study do not suggest that the EDEB/PRGF combination may provide a histomorphometric or medium-/long-term clinical advantage.

## Introduction

Sinus augmentation is a well-known approach to allow implant-supported rehabilitation in the atrophic posterior maxilla [1–7]. If the residual ridge height is smaller than 4 mm, the lateral approach is followed, involving elevating a vestibular flap, performing a lateral osteotomy to expose the sinus membrane, detaching it to reach the medial sinus wall, and grafting a bone substitute. Delayed implant placement follows. The ideal bone graft should be biocompatible, osteoconductive, osteogenic, and possibly stimulating the events that lead to bone regeneration, that is, osteoinductive [8, 9]. Autogenous bone displays all these features, yet its collection involves opening a second surgical site, increasing the patient’s discomfort and morbidity [10, 11]. Therefore, alternative grafts are used, including synthetic and natural bone substitutes. Among the latter, xenografts seem a viable alternative because of the similarity between the chemical composition and tridimensional structure of human and another mammal’s bone mineral portion [12]. Xenografts are manufactured processing heterologous bone to eliminate antigens that would lead to graft rejection [13, 14]. Processing may involve using high temperatures, leading to elimination of any organic bone component; this is the case of anorganic bovine bone [15], the graft most used in oral surgery [16, 17]. An alternative process based on using lytic enzymes working at lower temperatures (about 37 °C) is applied to manufacture enzyme-deantigenic equine bone (EDEB). This process, that involves selective degradation of antigens, allows preserving type I bone collagen intact [18]. Such EDEB feature was claimed to explain the greater amount of newly formed bone that could be observed when sinuses [18–20] and post-extraction sockets [18] were grafted with EDEB, compared to anorganic bovine bone, possibly for a different rate of osteoclast adhesion on the two biomaterials [21, 22]. While type I collagen may have some pro-regenerative effect, still EDEB lacks any osteogenic or other inductive potential. To possibly enhance regeneration, EDEB as well as other bone substitutes may be mixed with biological adjuncts, including cell extracts and preparations or blood derivatives, such as adipose-derived mesenchymal stem cells (ASCs), bone marrow aspirate or concentrate (BMA/BMC), platelet-rich plasma (PRP), leukocyte/platelet-rich fibrin (L-PRF or PRF); plasma-rich in growth factor (PRGF); autogenous fibrin (AF); magnesium-enriched hydroxyapatite (MHA); and others [23–32]. While published literature exists concerning the use of these adjuncts in combination with other xenografts in alveolar bone augmentation procedures, no evidence has been ever published concerning the use of any of them concomitantly to EDEB grafting. The authors have used EDEB in their clinical practices for some years, sometimes combining it with PRGF when performing lateral sinus augmentation surgeries and delayed implant placement. This study aims to provide a first retrospective assessment of the clinical and histomorphometric outcomes that could be observed following these interventions.

## Materials and methods

### Retrospective data collection

Clinical records were screened of consecutive patients who underwent lateral sinus augmentation surgeries and delayed implant placement at the private practice of one of the authors (DDS) between January 2008 and December 2012 and were then rehabilitated through an implant-supported prosthesis. Records were included for analysis if they concerned patients who (1) had sinuses grafted using EDEB (Osteoplant Osteoxenon Mix Granules, Bioteck, Arcugnano, Vicenza, Italy) in combination with PRGF; (2) had implants placed between 4 and 8 months from grafting; (3) had implants subjected to delayed loading; and (4) had at least one biopsy collected at the grafted sinuses at the time of implant placement. Other inclusion criteria were an age between 18 and 70 years and the lack of any systemic diseases. Patients were eligible for regenerative treatment if they did not present any of the following: pregnancy; osteoporosis, neoplasia, or psychiatric disease; acute oral infections; coagulation disorders; history of chemotherapy or radiotherapy in the head or neck region; immunocompromised status; current bisphosphonate therapy; chronic alcohol or drug abuse; or smoking more than 10 cigarettes per day. All patients provided their informed consent to the treatment, to biopsy collection at the time of implant placement, and to the use of their clinical data for retrospective collection and analysis. Smoking patients were asked to discontinue smoking one week prior to surgery and to not smoke three weeks thereafter. Biopsies were collected when preparing the implants seats, in correspondence to the sinuses being grafted; therefore, patients were not subjected to additional interventions other than those involved in standard, routine implant placement. All procedures performed in this study involving human participants were in accordance with the ethical standards of the institutional and/or national research committee and with the 1964 Helsinki declaration and its later amendments or comparable ethical standards. The local ethical committee approved the study.

### PRGF preparation, sinus grafting, and implant surgery

Peripheral blood (20–30 ml) was collected from each patient before surgery and placed into 5 ml tubes containing 3.8% (weight/vol) sodium citrate as an anticoagulant. Blood was centrifuged at 580 g for 8 min using a dedicate system (BTI, Vitoria, Spain). The plasma fraction was collected, excluding the buffy coat, and placed in a dish. The PRGF activator (BTI, Spain) was added in a 0.05:1 vol/vol activator/preparation ratio to initiate clotting and the formation of the fibrin matrix. Incubation at 37 °C for 30 min followed. To prepare the fibrin membrane, the plasma fraction located at the top of the tubes was transferred to a glass bowl.

Patients rinsed with chlorhexidine 0.2% (Corsodyl, Glaxo-SmithKline, Verona, Italy), to be continued for 2 weeks after surgery. Antibiotic prophylaxis (amoxicillin/clavulanic acid, Augmentin, Glaxo-SmithKline) (2 g, 1 h before surgery and then every 12 h for 8–10 days) was initiated. Nimesulide 100 mg (Aulin, Roche, Milano, Italy) was administered 1 h before surgery and then twice a day for 7 days. The surgical area was anesthetized with articaine hydrochloride 40 mg/mL and adrenaline 1:100,000. A full mucoperiosteal flap was elevated, and the vestibular bone wall exposed. Antrostomy was performed using a rotating bur, followed by an appropriate insert mounted on a piezoelectric instrument (Mectron, Carasco, Italy). The sinus membrane was gently detached and elevated up to the medial sinus wall using dedicated sinus elevation hand instruments (Hu-Friedy, Chicago, USA). EDEB 1:1 cancellous:cortical granules (Osteoplant Osteoxenon, Bioteck, Italy) were then mixed with the PRGF, in an approximately 1:1 vol/vol ratio, and the cavity under the membrane was then grafted using the mixture, applying a gentle pressure to stabilize it. The antrostomy was then protected using a collagen membrane (Biocollagen, Bioteck, Italy), covered with the fibrin membrane to enhance soft tissue healing, and the flap sutured using a 5-0 synthetic filament. Sutures were removed after 10 days. Patients were subsequently controlled once a month; an intra-oral digital radiograph was taken at each control visit. Approximately 4 to 7 months later, implants were placed as follows. Antibiotic prophylaxis and anesthesia were performed as in the extractive and grafting surgery, a full-thickness flap was elevated, and a biopsy sample was collected using a trephine, drilling the occlusal aspect of the alveolar ridge under irrigation. Biopsies were approximately 3 mm wide and 10 mm long and were marked on the occlusal side for orientation during histologic processing. Implants were double-etched, sandblasted fixtures (Xive, Dentsply, USA) varying from 3.75 to 5.0 mm in diameter and from 10 to 14 mm in length. Three months later, they were uncovered, and healing screws attached. Three weeks later, a radiograph was taken, and a dental impression was made using pick-up impression copings. A provisional prosthesis was manufactured and delivered after 10 days. Patients wore this for approximately 40 days, at which point the definitive abutments and metal-ceramic crowns were delivered. Patients followed a maintenance program comprising professional oral hygiene every 6 months.

### Histologic and histomorphometric analysis

Each biopsy was placed in a test tube containing buffered 10% formalin, after marking it at the coronal side using a dermographic pen. The tube was marked with a code that could not be related to the patient and sent to the histologic lab; histologists did not know, therefore, which patient the sample corresponded to. Bone cores were decalcified for 21 days in a 0.76 M/1.6 M sodium formate/formic acid solution (Panreac Quimica, Barcelona, Spain). The sample was subsequently dehydrated in ascending concentrations of ethanol and embedded in paraffin. Bone cores were cut into 5-μm-thick sections, mounted on slides, and stained with hematoxylin-eosin. Qualitative examination followed, aimed at identifying any sign of inflammatory or immune reactions. Morphometrical measurements were performed on whole sample digital photomicrographs collected at × 10 magnification using the Image J 1.33 analysis software (National Institute of Health, Bethesda, USA). For each image, the total sample area (TSA), the total bone area (TBA), the newly formed bone area (LBA), and the residual bone substitute area (RBA) were measured. Newly formed bone could be distinguished because of its lighter, purple color, indicating greater affinity to eosin than the residual biomaterial (which appeared blue-violet and darker)—possibly because of its newly-formed bone collagen content. When marking the sample area, the histologist excluded the patients’ own residual bone, if preserved during collection: this was easily recognizable at the coronal side of the biopsy as a homogenous bone layer not containing any graft. Each assessment was repeated in triplicate. Average newly formed bone (NFB) and residual biomaterial (RB) were then calculated and expressed as the percentage over the total sample area (%NFB = LBA × 100/TSA; %RB = RBA × 100/TSA).

### Implant success and peri-implant marginal bone loss

**Fig. 1:**
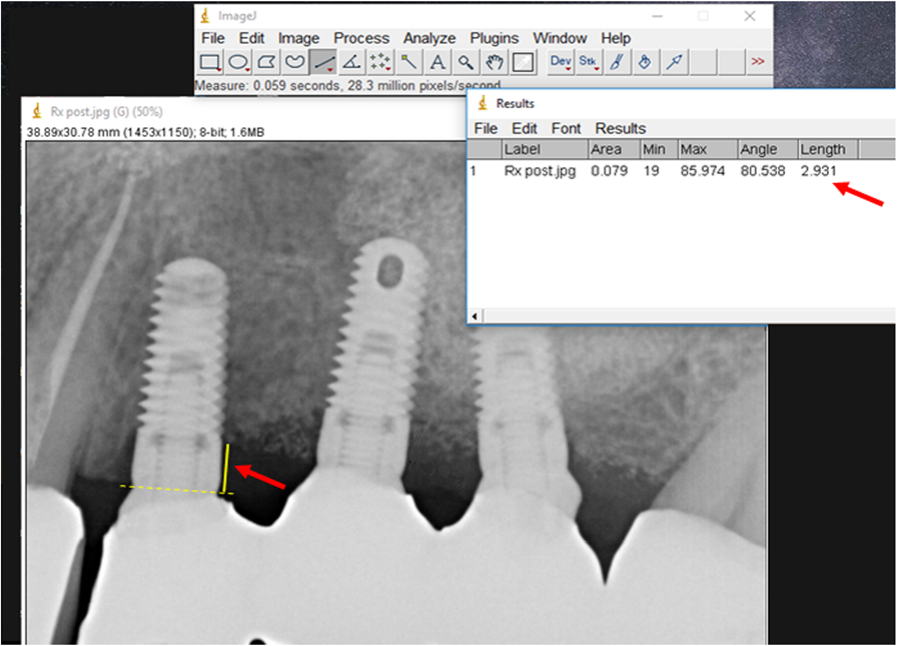
Measuring peri-implant bone levels on intraoral radiographs. The peri-implant bone level at the distal side of an implant is measured by drawing a line (yellow, dotted) over the implant-abutment interface, and measuring its distance (yellow, full; indicated by the red arrow in the radiograph) from the most apical point of crestal bone in intimate contact with the implant through to the calibrated image-analysis software (in the example being shown, 2.931 mm, indicated by the red arrow in the measurement window). The measurement will be repeated at the mesial side, and the two will be averaged. The MBL will be calculated as the difference between this averaged value and that measured repeating the whole procedure on the corresponding immediate post-insertion intraoral radiograph

For all included patients, intraoral radiographs were taken digitally and analyzed using an image analysis software (ImageJ, NIH, Bethesda, US). This was calibrated using the known implant diameter at the most coronal portion of the implant neck. A single peri-implant marginal bone level was calculated by averaging the distances, at the mesial and distal implant sides, from the implant-abutment interface to the most apical point of crestal bone observed to be in intimate contact with the implant (Fig. 1). Accuracy of measurements was 0.01 mm. The marginal bone loss (MBL) for that implant at a given time-point was then calculated as the difference between the peri-implant bone level at that time point and that at implant insertion.

Implant success was evaluated according to criteria by Albrektsson and Zarb [33] including (i) absence of persistent pain, dysesthesia, or paresthesia in the implant area; (ii) absence of peri-implant infection with or without suppuration; (iii) absence of perceptible mobility of the implant; and (iv) absence of peri-implant bone resorption greater than 1.5 mm during the first year of loading and 0.2 mm/year in the following years. Implants were considered successful when all the above-mentioned conditions were met. Implants were also considered as failed if bone loss greater than half the implant length was observed on radiographs or if the implant showed mobility.

### Statistical analysis

Patients’ demographics were analyzed by means of descriptive statistics. NFB and RB data were summarized by providing their mean and standard deviations, after checking for data normality through Shapiro-Wilks tests. Differences between MBL over time were investigated by ANOVA tests, followed by post-hoc paired comparison tests (Bonferroni), again after checking for data normality by means of a Shapiro-Wilk test. Differences were regarded as significant if *p* < 0.05. A dedicated software program (Origin 2020, Microcal, Northampton, MA, USA) was used for all statistical analyses.

## Results

**Fig. 2:**
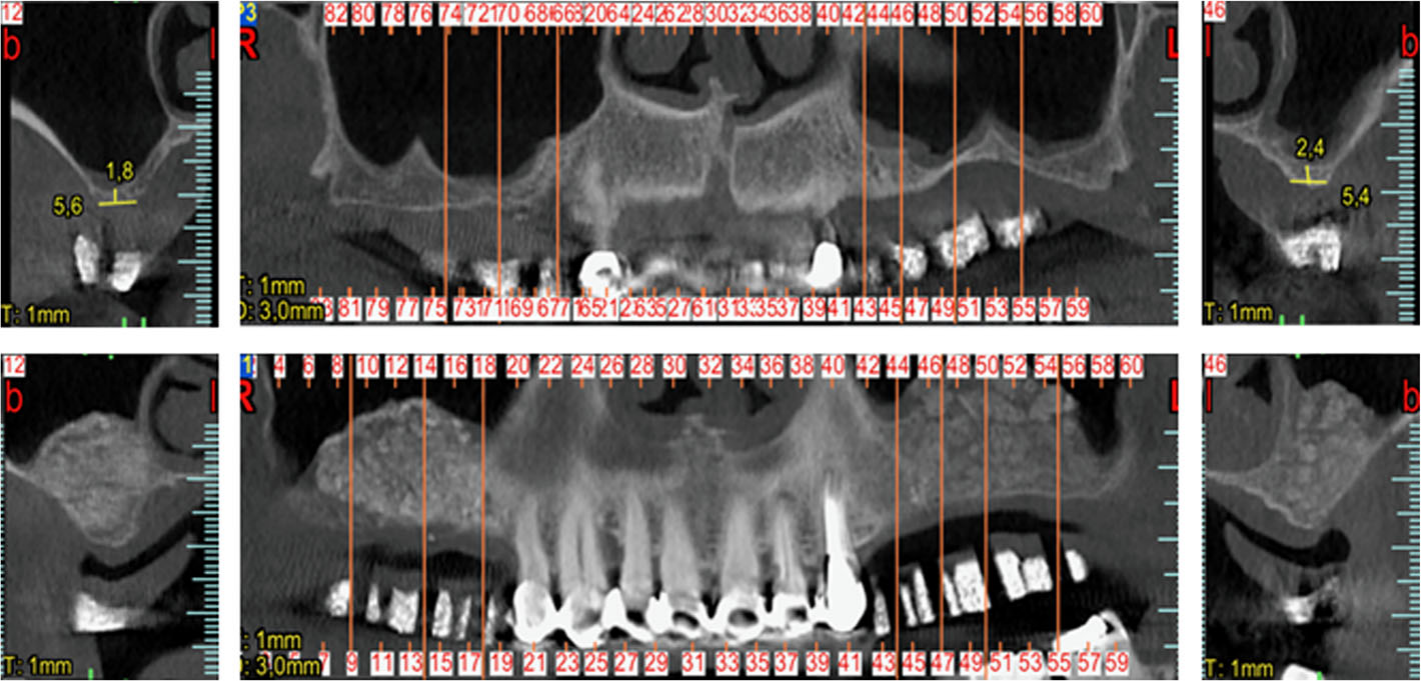
CBCT scans of the bilateral case depicted in Fig. 3, before (top) and after (bottom) the sinus augmentation surgery

**Fig. 3:**
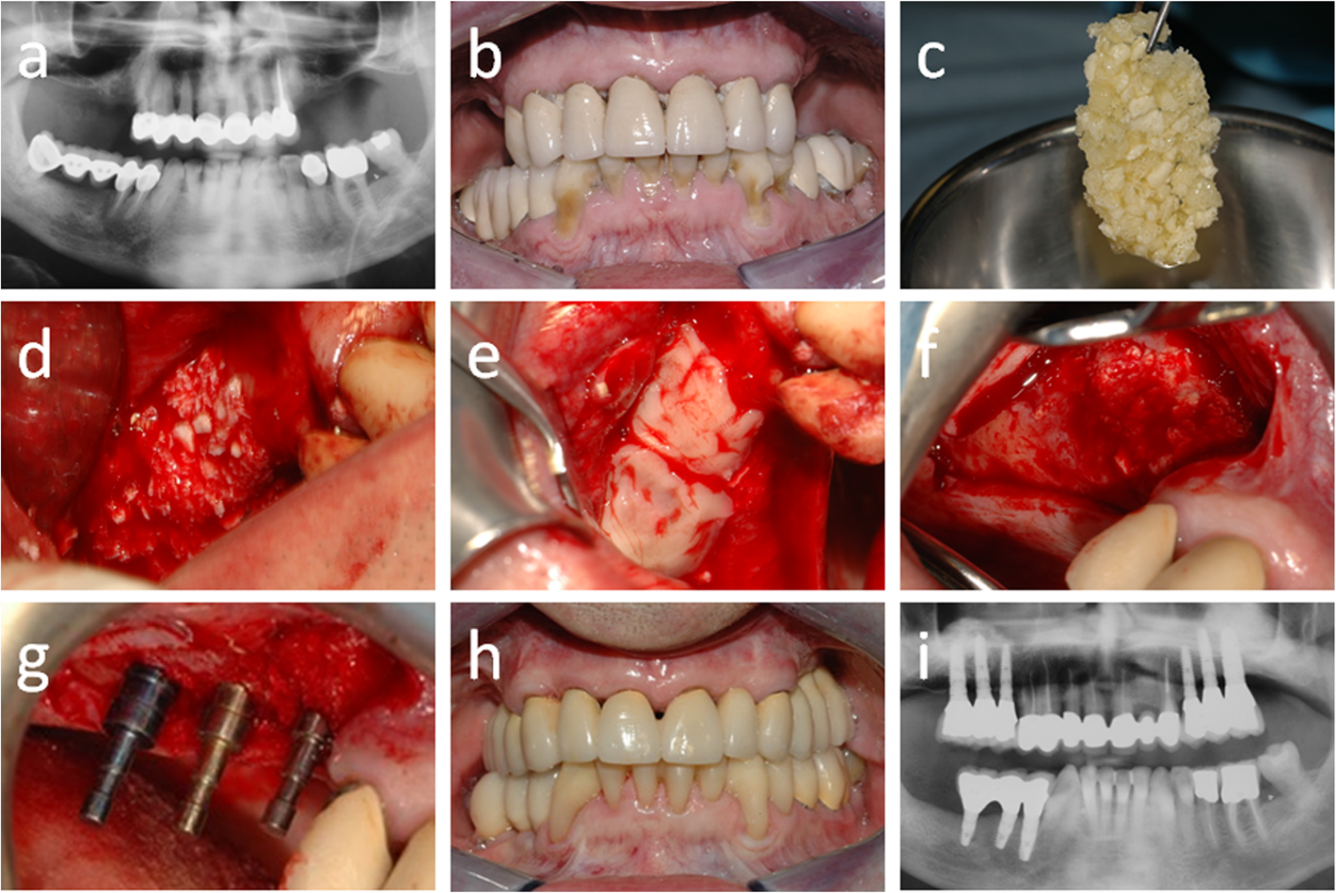
A representative clinical case (Patient #6, bilateral sinus augmentation). **a** The pre-operative panoramic X-ray. **b** The clinical appearance at presentation. **c** The EDEB/PRGF mixture. **d** The augmentation is complete. **e** The antrostomy is sealed using the fibrin membrane. **f** The bone ridge 4 months later at **g** implant placement. **h** The definitive rehabilitation. **i** 50-month control panoramic X-ray

**Fig. 4:**
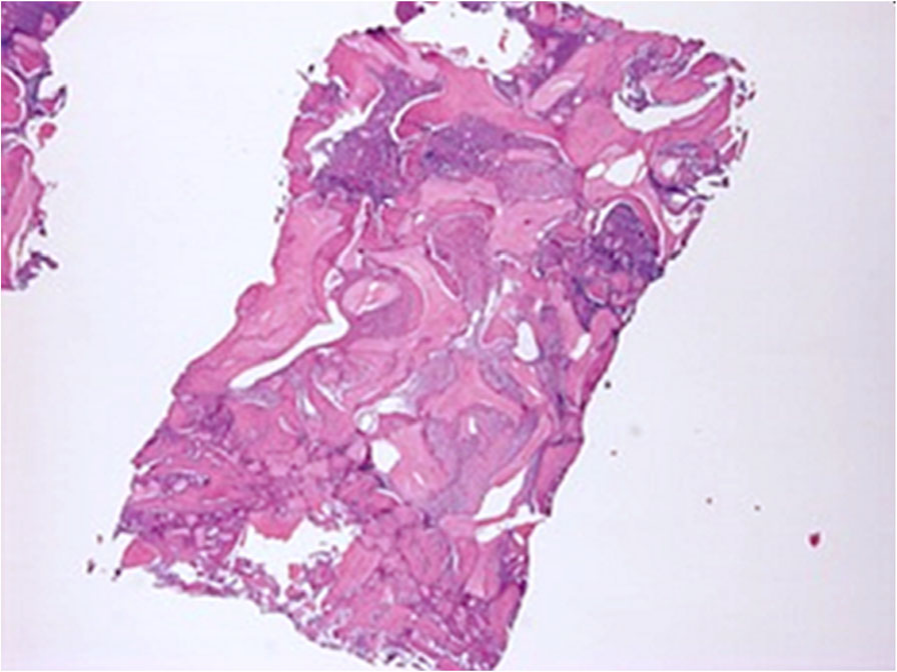
Histologic of a biopsy taken from the case shown in Figs. 2 and 3 (position 17). × 10 magnification. Particles of residual biomaterial (darker, blue-violet areas) are embedded within newly formed bone (lighter, purple areas)

**Fig. 5:**
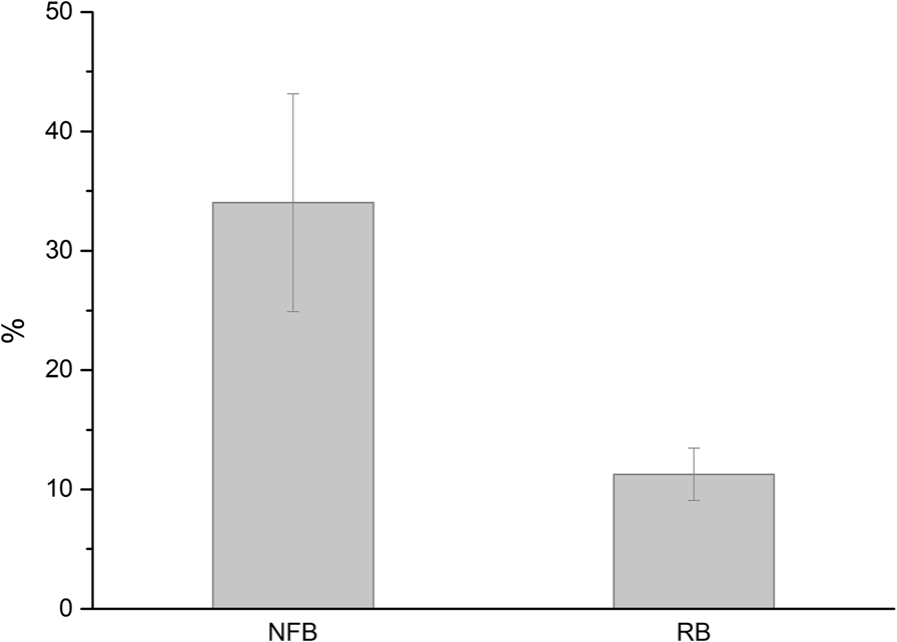
Average newly formed bone (NFB, %) and residual biomaterial (RB, %) 5.62 ± 1.13 months after grafting

**Fig. 6:**
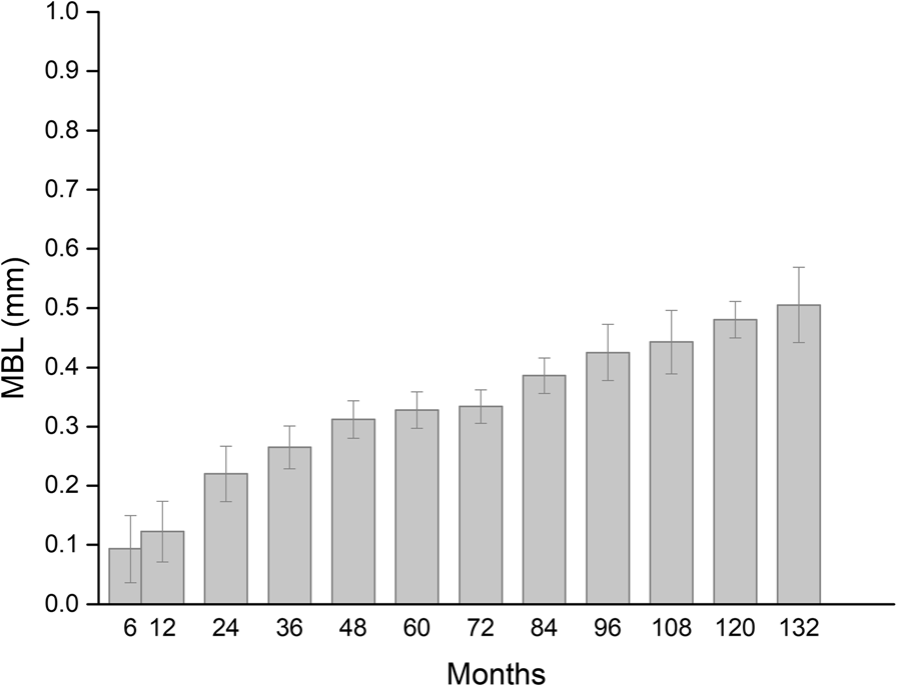
MBL (marginal bone loss, mm) over time, following final prosthesis delivery, of implants placed within sinuses augmented using the EDEB/PRGF mixture

Records concerned 11 patients, 6 (54.5%) women and 5 (45.5%) men, whose average age was 58.7 ± 8.3 years (range, 45–68), and who were followed for 86.5 ± 4.3 months (range, 50–133). Overall, 14 sinuses were augmented as two patients underwent bilateral sinus augmentation. Data concerning patient demographics, the sinuses being grafted, the healing and follow-up time, as well as implants and biopsies are shown in Table 1. Implants placed were 34, and as many biopsies were collected. The average healing time was 5.6 ± 1.1 months. No patient experienced intra-surgical or post-surgical complications. All patients were rehabilitated using screwed metal-ceramic prostheses. An illustrative case is depicted in Figs. 2 and 3; a histologic picture of a biopsy collected by the same patient is provided in Fig. 4. No signs of inflammation or immune reaction were observed concerning any biopsy. Considering the bone biopsy as the statistical unit of analysis, NFB was 34.0 ± 9.1% and RB was 11.3 ± 2.2% (*n* = 34, Fig. 5). Similar values were observed when the statistical unit of analysis was the patient (NFB, 34.9 ± 9.6%; RB: 11.2 ± 2.7%, *n* = 11). MBL of implants varied, after the final prosthesis delivery, as shown in Fig. 6. At the final follow-up, the implant survival rate was 100%, and no prosthetic failures were recorded. Both at the implant level (*n* = 34) and at the patient level (*n* = 11), the average MBL at the last follow-up was 0.40 ± 0.07 mm.

**Table 1:** The study data. Healing time (months) is the time from grafting surgery to implant placement. Follow up (months) is the time of the last control visit from definitive implant loading

Patient #	Gender	Age (years)	Tooth	Healing time (months)	Implant diameter (mm)	Implant length (mm)	NFB (%)	RB (%)	Follow-up (months)	MBL at final follow-up (mm)
1	F	56	16	4.5	4.5	13	35.25	8.45	133	0.55
			17	4.5	3.8	11	34.25	9.56	133	0.46
2	F	57	25	5.2	3.8	13	43.25	12.45	84	0.40
			26	5.2	4.5	11	42.75	12.48	84	0.38
			27	5.2	4.5	11	45.65	13.12	84	0.40
3	F	45	26	7	3.8	11	24.58	9.90	72	0.33
			27	7	3.8	9.5	26.89	9.56	72	0.34
4	F	67	15	6.5	4.5	13	38.47	12.25	86	0.38
			16	6.5	4.5	11	35.25	13.56	86	0.42
			17	6.5	4.5	11	39.25	11.20	86	0.38
			25	7.5	3.8	11	26.89	12.45	86	0.43
			26	7.5	5.5	9.5	30.15	10.10	86	0.40
5	F	57	15	4.5	3.8	13	44.25	15.15	73	0.34
			16	4.5	3.8	13	40.12	8.96	73	0.37
6	F	67	15	4	5.5	13	25.25	10.10	50	0.26
			16	4	4.5	11	23.65	12.25	50	0.32
			17	4	4.5	11	27.89	14.55	50	0.31
			25	5	3.8	13	33.25	10.10	50	0.33
			26	5	4.5	11	32.45	10.45	50	0.32
7	M	56	26	4.5	3.8	13	51.24	14.52	56	0.33
			27	4.5	4.5	11	58.89	13.22	56	0.35
8	M	45	15	7	5.5	13	20.56	10.90	122	0.48
			16	7	4.5	13	22.45	9.90	122	0.50
			17	7	4.5	9.5	26.45	12.25	122	0.52
9	M	67	25	6	3.8	13	45.25	15.52	98	0.44
			26	6	3.8	11	40.25	14.20	98	0.45
			15	6	4.5	11	32.29	10.23	98	0.45
			16	6	4.5	11	30.21	9.89	98	0.42
10	M	61	15	4.5	5.5	13	25.54	10.56	85	0.36
			16	4.5	4.5	11	24.56	12.45	85	0.40
			17	4.5	4.5	11	22.22	8.56	85	0.37
11	M	68	25	6.5	4.5	13	40.28	7.57	109	0.48
			26	6.5	4.5	11	37.19	6.59	109	0.45
			27	6.5	3.8	11	30.25	10.24	109	0.49
Mean		**58.73**		**5.62**			**34.03**	**11.27**	**86.47**	**0.40**
SD		**8.33**		**1.13**			**9.13**	**2.20**	**24.30**	**0.07**

*SD* standard deviation, *NFB* newly formed bone, *RB* residual biomaterial, *MBL* marginal bone loss

## Discussion

This study is the first providing histomorphometric and clinical data concerning the concomitant use of EDEB and PRGF for performing sinus augmentation procedures. As no patient experienced intra- or post-surgical complications, observations of the present study indicate that the concomitant use of EDEB and PRGF is safe. Safety of this combination is also supported by the lack of any inflammatory or immune reaction sign in any biopsy. Results of the present study may be discussed at the light of previous published data, within the limitation of not having direct access to original data from publications of other groups. Concerning grafting sinuses using EDEB alone, in a randomized clinical trial aimed at comparing anorganic bovine bone and EDEB in sinus augmentations procedures, 6 months after grafting EDEB NFB was 46.9 ± 12.8% and RB was 11.1 ± 9.3% [19]; in a retrospective study aimed at investigating the kinetic of new bone formation when EDEB was used in sinus augmentation procedures, NFB was observed to vary, according to the time from surgery, between 38.4 ± 14.2% (3–5 months) and 37.8 ± 14.7% (6–8 months) and RB varied between 9.2 ± 6.6% (3–5 months) and 11.7 ± 8.4% (6–8 months). All these values may be regarded as overlapping those of the present study within the variability of the experimental data. Prospective investigations should therefore be performed to confirm this preliminary hypothesis, that is, that adding PRGF to EDEB seems to not have provided any increase in NFB that could be observed at the time of implant placement. The possibility that adding PRGF to carry out sinus augmentation will not result in increased new bone formation is tenable, as results concerning the use of PRGF, when used concomitantly to other bone grafts to perform sinus augmentation procedures, are not conclusive concerning newly formed bone. Anitua et al. [34] when performing lateral sinus augmentations using a mixture of PRGF and anorganic bovine bone found, 5–6 months after grafting, an amount of NFB equal to 25.2 ± 4.6% (*n* = 8); such amount looks similar to that observed in studies where anorganic bovine bone was used alone and biopsies were collected 5–6 months after grafting (Di Stefano et al. [19]: NFB, 25.1 ± 7.3%, *n* = 20; Schmitt et al. [35]: NFB, 26.0 ± 5.2%, *n* = 12; Testori et al. [36]: 18.8 ± 4.7%, *n* = 11; Iezzi et al. [37]: 32.9 ± 0.5%, *n* = 12; Ferreira et al. [38]: 39.0% ± 12.0%, *n* = 7; Cordaro et al. [39]: NFB, 19.8 ± 7.9%, *n* = 18). Concerning the few split-mouth studies published at present comparing anorganic bovine bone alone to its mixture with PRGF, Anitua et al. [40] reported anecdotic data concerning two patients whose NFB, in their sinuses grafted with the mixture was similar to that observed, in other studies, when sinuses are grafted with anorganic bone alone and, surprisingly, it was much lower when anorganic bovine bone was grafted alone (21.4% vs. 8.4% and 28.4% vs. 8.2%). Batas et al. [41] in 5 bilateral sinus augmentation cases found that NFB was 37.8 ± 3.1% with anorganic bovine bone and 35.6 ± 8.3% with the mixture, the difference being not statistically significant. Overall, published data concerning PRGF and anorganic bovine bone suggest adding PRGF provides no or little advantage as far as NFB in lateral sinus augmentation is concerned, as outlined also in a recent systematic review on the matter [30]. The results of the present study concerning mixing EDEB with PRGF seem to be consistent with such findings.

Current published evidence on using PRGF in sinus augmentation does not answer the question if the mixture may accelerate bone formation in the early stages after grafting, and if, accordingly, implant insertion could be carried out earlier; this should be the subject of appropriately designed prospective studies. Such advantage might be of a certain importance when the bone substitute being grafted is known to undergo slow remodeling, as with anorganic bovine bone [18, 19]. Concerning EDEB, previous findings [20] show that it already allows early bone formation, to such extent that NFB in biopsies collected 3–5 months after grafting is not significantly different from that of biopsies collected later, up to 12 months from grafting. Accordingly, adding PRGF to this specific bone substitute might be of no advantage at all. Current published evidence does not also answer the question if adding PRGF to any bone substitute when performing lateral sinus augmentation may decrease the variability (i.e., the ratio between the standard deviation and the average) of NFB. Current studies, in fact, dealt with a too low number of biopsies, and prospective investigations involving a greater number of samples should be undertaken to answer this question, as well as the question if adding PRGF might be advantageous for specific sub-sets of patients.

Results of the present study concerning medium-term implant MBL and implant and prosthetic success can be regarded, within the limit of the small number of implants and subjects being investigated, consistent with those concerning implant-supported rehabilitations delivered to patients whose sinuses were augmented either with EDEB or with other bone substitutes, either mixed with PRGF or not [30, 34, 42–45]. Thus, mixing EDEB and PRGF for performing sinus augmentations seems to not provide any long-term advantage concerning these outcomes. The authors confirm that patients of the present study seemed to show better soft tissue healing than those that, in their clinical practice, were not—in accordance with some published results [30, 46]. This, though, was not an endpoint of the present study and further studies should be carried out to investigate how this endpoint is affected by the EDEB/PRGF combination. The authors also appreciated the enhanced handling properties of the particulate/PRGF mixture other authors have already highlighted [30, 34, 40, 41]. Major limitations of the present study are its retrospective design and the limited number of subjects and implants it involved; further, it lacked a control group. Limitations also include not assessing other endpoints of interests, including bone gain at grafted sinuses; this should be addressed by appropriate studies, aimed to compare sinuses grafted with EDEB alone to ones grafted by the PRGF-EDEB mixture to assess if any difference in bone gain can be observed. Further, using different bone analysis techniques, including assessing non-decalcified biopsies, might have provided additional insights concerning the effect of adding PRGF to this biomaterial, including allowing semi-quantitative or quantitative assessment of inflammation or other adverse reactions, which could not be carried out reliably on decalcified samples. Patients smoking less than 10 cigarettes a day were included in the present study to avoid introducing a too strong data selection bias; prospective studies should instead carefully define appropriate and stringent inclusion criteria to minimize the effect of confounding variables on the outcomes of interest. Given all these limitations, results of the present study should be regarded as altogether preliminary and indicative, and findings be subjected to verification through prospective, comparative (split-mouth) studies involving a greater number of subjects.

## Conclusions

Within the limitations of the present study, grafting EDEB/PRGF for lateral sinus augmentation and delayed implant placement seems to be safe. Compared to published data concerning EDEB alone, results of the present study do not suggest that the EDEB/PRGF combination may provide a histomorphometric or medium/long term clinical advantage.

## Data Availability

Not applicable.
